# Social support and adolescent mental health in Kenya: a parallel mediation analysis of perceived control and gratitude

**DOI:** 10.3389/fped.2025.1626249

**Published:** 2025-08-22

**Authors:** Ming Zhao, Heng Miao, Li-Li Zhu, Xiao-Han Zhang, Li-Wei Zang

**Affiliations:** ^1^ Department of Psychiatry, Wenzhou Seventh People’s Hospital, Wenzhou, China; ^2^ School of Mental Health, Wenzhou Medical University, Wenzhou, China; ^3^ Career Education Research Center, Wenzhou University, Wenzhou, China; ^4^ Renji College, Wenzhou Medical University, Wenzhou, China

**Keywords:** social support, adolescent mental health, perceived control, gratitude, Kenyan adolescents

## Abstract

**Introduction:**

Adolescent mental health is a global concern. While social support is a known protective factor, the mechanisms through which it impacts mental health, particularly in diverse settings like Kenya, require further exploration. This study aimed to investigate how perceived control and gratitude mediate the relationship between social support and mental health (symptoms of depression and anxiety) among Kenyan adolescents.

**Methods:**

A sample of 1,674 adolescents (aged 13–18) from four secondary schools completed validated measures of social support (MSPSS), perceived control (PCS), gratitude (GQ-6), depression (PHQ-8), and anxiety (GAD-7). The data were analyzed using structural equation modeling, controlling for age and gender.

**Results:**

Social support was directly associated with lower levels of depression (*β* = −023) and anxiety (*β* = −023). Social support also positively predicted perceived control (*β* = 026) and gratitude (*β* = 049), both of which were, in turn, linked to lower depression and anxiety. Crucially, significant indirect effects were found, demonstrating that social support reduced symptoms of depression and anxiety through the parallel mediating pathways of both perceived control (depression: *β* = −005; anxiety: *β* = −004) and gratitude (depression: *β* = −008; anxiety: *β* = −006).

**Discussion:**

The findings highlight that perceived control and gratitude are crucial mechanisms through which social support benefits adolescent mental health in Kenya. These psychological resources function as parallel pathways linking social connections to well-being. Interventions should therefore be designed not only to bolster social support systems but also to concurrently cultivate adolescents' sense of personal control and gratitude.

## Introduction

Adolescence is a critical developmental period characterized by profound physical, psychological, and social transformations, with significant implications for mental health (1). Globally, mental health conditions constitute a major disease burden among young people, and the onset of many adult psychiatric disorders can be traced to these formative years (2). Consequently, identifying and promoting factors that bolster adolescent well-being is a public health priority. Among these factors, social support has been consistently identified as a crucial protective resource (3). However, the mechanisms through which social support exerts its beneficial effects, particularly in diverse cultural contexts such as Kenya, are still being elucidated. This study focuses on the roles of perceived control and gratitude as potential psychological mediators linking social support to mental health outcomes—specifically depression and anxiety symptoms—among Kenyan adolescents. Investigating these pathways is vital for developing culturally sensitive and effective interventions aimed at bolstering adolescent resilience and mental health in under-resourced settings.

### Theoretical framework

Our theoretical framework is anchored in the Stress-Buffering Hypothesis, which posits that social support directly mitigates the negative effects of stress on mental health (4). We extend this model by proposing two parallel pathways through which social support also builds internal psychological resources. The first, a cognitive-agentic pathway, is informed by Social Cognitive Theory, arguing that support enhances perceived control by bolstering self-efficacy (5). The second, an affective-appreciative pathway, draws on the Broaden-and-Build Theory, suggesting that support elicits gratitude, a positive emotion that builds lasting psychological resilience (6). This integrated framework explains how social support not only protects against distress but also proactively fosters the cognitive and affective resources essential for well-being.

### Social support, mental health, and potential mediators

Social support, defined as the perception or experience of being cared for, loved, esteemed, and part of a mutually supportive social network (7, 8), is a cornerstone of psychological well-being across the lifespan. For adolescents, support from various sources, including family, friends, and significant others—as captured by measures like the Multidimensional Scale of Perceived Social Support (MSPSS) (9)—is particularly salient. A vast body of research demonstrates that higher levels of perceived social support are associated with lower levels of internalizing symptoms, such as depression and anxiety, in adolescents (10). The stress-buffering hypothesis, for instance, posits that social support can protect individuals from the pathogenic effects of stress by providing resources, encouragement, or alternative appraisals of stressful situations (11).

Beyond its direct effects, social support may also foster other positive psychological resources that, in turn, promote mental health. One such resource is perceived control, which refers to an individual's belief in their capacity to influence outcomes and achieve desired goals through their own efforts (12). The Perceived Control Scale (PCS) (13), particularly its academic domain subscale used in this study, taps into these agentic beliefs. Adolescents with a stronger sense of perceived control tend to exhibit better coping skills (14), higher academic achievement (15), and lower levels of psychological distress, including depression and anxiety (16). Socially supportive environments can cultivate perceived control by providing opportunities for mastery, offering encouragement, and reinforcing beliefs in one's capabilities (5). Thus, social support may indirectly alleviate mental health problems by enhancing an adolescent's perceived control.

Another critical psychological resource that may link social support to mental health is gratitude. Gratitude, often conceptualized as a disposition to recognize and appreciate the positive aspects of life, has emerged as a key construct in positive psychology. Assessed using tools like the Gratitude Questionnaire-6 (GQ-6) (17), higher levels of gratitude in adolescents are consistently linked to greater life satisfaction, optimism, and prosocial behavior, as well as lower levels of depression and anxiety; these benefits are supported by recent meta-analyses showing gratitude interventions improve mental health and reduce anxiety and depression (18). Fredrickson's broaden-and-build theory of positive emotions suggests that gratitude, as a positive emotion, can broaden an individual's thought-action repertoire and build enduring personal resources, including social bonds and coping skills (19). Experiencing social support may naturally elicit feelings of gratitude, as individuals recognize the kindness and care received from others (20). This heightened sense of gratitude can then contribute to improved mental health by fostering positive affect and resilient coping mechanisms (21).

While the individual contributions of social support, perceived control, and gratitude to adolescent mental health are well-documented, the interplay between these factors, particularly their roles as parallel mediators, is less explored. The Patient Health Questionnaire-8 (PHQ-8) (22) and the Generalized Anxiety Disorder Screener-7 (GAD-7) (23) are widely used and validated measures to assess depressive and anxiety symptoms, respectively, and understanding how psychosocial resources collectively impact these outcomes is crucial. Research in various contexts has begun to explore such mediational pathways, including how gratitude can mediate the relationship between social support and well-being (24). However, there is a pressing need to examine these relationships in diverse cultural settings. Adolescent mental health in Sub-Saharan Africa, including Kenya, faces unique challenges, including poverty, educational pressures, and limited access to mental health services, with recent systematic reviews continuing to highlight high prevalence rates of mental health problems among youth in the region (25). Understanding the protective factors within these specific socio-cultural contexts is paramount for designing effective, locally relevant interventions.

### Gaps in the literature

Despite the established links between social support and adolescent mental health, several gaps remain in the literature that the current study aims to address. Firstly, much of the existing research on the psychological mechanisms linking social support to mental health outcomes has been conducted in Western, Educated, Industrialized, Rich, and Democratic (WEIRD) societies (26). There is a significant need for studies that examine these processes in non-Western cultural contexts, such as Kenya. In these contexts, culture influences what is considered a mental health problem, how it is understood, the expression and impact of social support, and the kind of practical solutions considered appropriate (27). Indeed, research highlights a continued scarcity in investigations into the symptomatology and correlates of common mental health conditions among Kenyan adolescents (28). Understanding these dynamics in Kenyan adolescents is crucial for tailoring interventions that are culturally congruent and effective.

Secondly, while perceived control and gratitude have independently been recognized as important correlates and mediators of mental health in relation to social support (29, 30), few studies have simultaneously investigated their roles as parallel mediators in the relationship between social support and mental health outcomes (specifically depression and anxiety symptoms) among adolescents. Examining these mediators in parallel allows for a more comprehensive understanding of the multiple pathways through which social support may exert its protective effects. This approach can help identify whether certain psychological resources are more potent mediators or if they operate in concert to influence mental well-being. The current study's use of structural equation modeling to test a parallel mediation model addresses this complexity.

Finally, the specific combination of perceived control (an agentic, cognitive belief) and gratitude (an affective, appreciative disposition) as co-occurring mediators of social support's impact on both depression and anxiety offers a nuanced perspective. It acknowledges that social support can bolster not only an adolescent's sense of capability but also their positive emotional orientation towards life. The present research, by focusing on Kenyan adolescents and employing a robust analytical approach to examine these parallel mediation pathways, seeks to provide valuable insights into the complex interplay of social, cognitive, and affective factors in shaping adolescent mental health.

### Research objectives and hypotheses

Based on theoretical framework and the literature review, the primary objectives of this study to fit the gaps mentioned above: (1) To examine the direct associations between perceived social support and symptoms of depression and anxiety among Kenyan adolescents; (2) To investigate the mediating roles of perceived control and gratitude, in parallel, in the relationships between social support and symptoms of depression and anxiety.

In addition, we hypothesized that:

(1) H1: Perceived social support would be negatively associated with symptoms of depression and anxiety.
(2) H2: Perceived social support would be positively associated with perceived control and gratitude.
(3) H3: Perceived control and gratitude would be negatively associated with symptoms of depression and anxiety.
(4) H4: Perceived control would significantly mediate the relationship between social support and symptoms of depression and anxiety.
(5) H5: Gratitude would significantly mediate the relationship between social support and symptoms of depression and anxiety.

## Method

### Participants and procedure

The initial study sample was drawn from four secondary schools in Kenya. The selection of these schools was purposeful, designed to capture a diversity of student experiences based on school type and location. Specifically, the sample included two high-resource national boarding schools (one for boys, one for girls) in the urban Nairobi County, and two lower-resource sub-county schools (one all-girls day school, one mixed-gender day school) in the neighboring peri-urban Kiambu County. This approach allowed for the inclusion of adolescents from varied socioeconomic and academic environments. It is important to note that the sample is not nationally representative of all Kenyan adolescents, as it was drawn from two of Kenya's 47 counties. The study protocol was approved by the Maseno University Ethics Review Committee (MSU/DRPI/MUERC/00727/19). Informed consent was obtained from the guardians of all participants, and informed assent was secured from participants who were minors at the time of data collection. For the current analysis, we focused on adolescents between 13 and 18 years of age. Cases with missing data on key variables were removed using listwise deletion.

### Measures

#### Social support

Social support was measured using the Multidimensional Scale of Perceived Social Support (MSPSS) (9). The MSPSS consists of 12 items designed to assess perceptions of support from family, friends, and significant others. Participants rated their agreement with statements on a 7-point Likert scale. Higher total scores indicate greater perceived social support. In the current sample, the MSPSS demonstrated good internal consistency (*α* = .881).

#### Perceived control

The Perceived Control Scale (PCS) (13) was used to assess participants’ beliefs about their ability to obtain desired outcomes and avoid undesired outcomes through their own efforts. The academic domain subscale consisting of 8 items was used in this study. Items were rated on a 4-point scale, with higher mean scores indicating greater perceived control. The internal consistency for the PCS in the current sample was good (*α* = .800).

#### Gratitude

Gratitude was assessed using the brief Gratitude Questionnaire-6 (GQ-6) (17). This 6-item measure evaluates participants’ disposition to experience gratitude in daily life. Items were rated on a 7-point Likert scale. Items 3 and 6 were reverse-scored, with higher total scores indicating greater dispositional gratitude. The internal consistency for the GQ-6 in the current sample was acceptable (*α* = .783).

#### Depression symptoms

Depressive symptoms were measured using the Patient Health Questionnaire-8 (PHQ-8) (22), an 8-item screening tool that assesses the frequency of depressive symptoms over the past two weeks. Items were rated on a 4-point scale (0 = not at all to 3 = nearly every day). Higher total scores indicate more severe depressive symptoms. Internal consistency for the PHQ-8 in the current sample was acceptable (*α* = .770).

#### Anxiety symptoms

Anxiety symptoms were assessed using the Generalized Anxiety Disorder Screener-7 (GAD-7) (23), a 7-item measure of anxiety symptom severity over the past two weeks. Items were rated on a 4-point scale (0 = not at all to 3 = nearly every day). Higher total scores indicate more severe anxiety symptoms. The GAD-7 demonstrated good internal consistency in the current sample (*α* = .826).

### Data analysis

We conducted a parallel mediation analysis using observed-variable path analysis within the structural equation modeling (SEM) framework, using the lavaan package (31) in R to examine the direct and indirect relationships between social support, perceived control, gratitude, and mental health outcomes (depression and anxiety symptoms). Missing data were handled through listwise deletion, and only participants with complete data on all study variables (*N* = 1,674) were included in the analysis. The hypothesized model specified social support as the predictor variable, perceived control and gratitude as parallel mediators, and depression and anxiety symptoms as outcome variables. Direct pathways from social support to both depression and anxiety were specified, as well as indirect pathways through perceived control and gratitude. The model also included covariances between the two mediators (perceived control and gratitude) and between the two outcomes (depression and anxiety). We used the robust maximum likelihood (MLR) estimator to account for potential non-normality in the data. The significance of indirect effects was tested using bootstrapping (implicitly via MLR standard errors in the summary output) to generate 95% confidence intervals. Standardized coefficients were reported to facilitate interpretation of effect sizes. To examine the robustness of the findings, we also tested an alternative model that controlled for demographic variables (age and gender). Model comparison was performed using the Akaike Information Criterion (AIC) and Bayesian Information Criterion (BIC), with lower values indicating better fit. The specified path analysis model utilized observed variables for all constructs, resulting in a saturated model with zero degrees of freedom.

## Results

### Preliminary analyses

The initial sample from the dataset of 2,192 adolescent students (aged 12–19; 57.57% female). For the current analysis, we focused on adolescents between 13 and 18 years of age. After removing cases with missing data on key variables, the final analytic sample comprised 1,674 participants. Table 1 presents the descriptive statistics and bivariate correlations among the primary study variables. On average, participants reported moderate levels of social support, perceived control, and gratitude. As expected, social support, perceived control, and gratitude were each moderately and negatively correlated with both depression and anxiety symptoms (rs ranging from −0.25 to −0.36, all *p* < .001). These findings align with previous literature, suggesting that greater perceived social support and positive psychological resources are associated with better mental health outcomes among adolescents.

**Table 1:** Descriptive statistics and bivariate correlations among study variables (*N* = 1,674).

Variable	M	SD	1	2	3	4	5
1. Social support (MSPSS)	61.72	13.52	—				
2. Perceived control (PCS)^a^	2.75	0.41	.26***	—			
3. Gratitude (GQ-6)	35.33	5.96	.49***	.37***	—		
4. Depression (PHQ-8)	7.90	5.17	−.36***	−.32***	−.35***	—	
5. Anxiety (GAD-7)	7.42	5.12	−.33***	−.25***	−.29***	.71***	—

Note: M, mean; SD, standard deviation; MSPSS, multidimensional scale of perceived social support; PCS, perceived control scale; GQ-6, gratitude questionnaire-6; PHQ-8, patient health questionnaire-8; GAD-7, generalized anxiety disorder-7.

^a^ Perceived control score represents the mean of item responses, whereas other scales represent total summed scores.

*** *p* < .001.

### Mediation analysis

**Figure 1:**
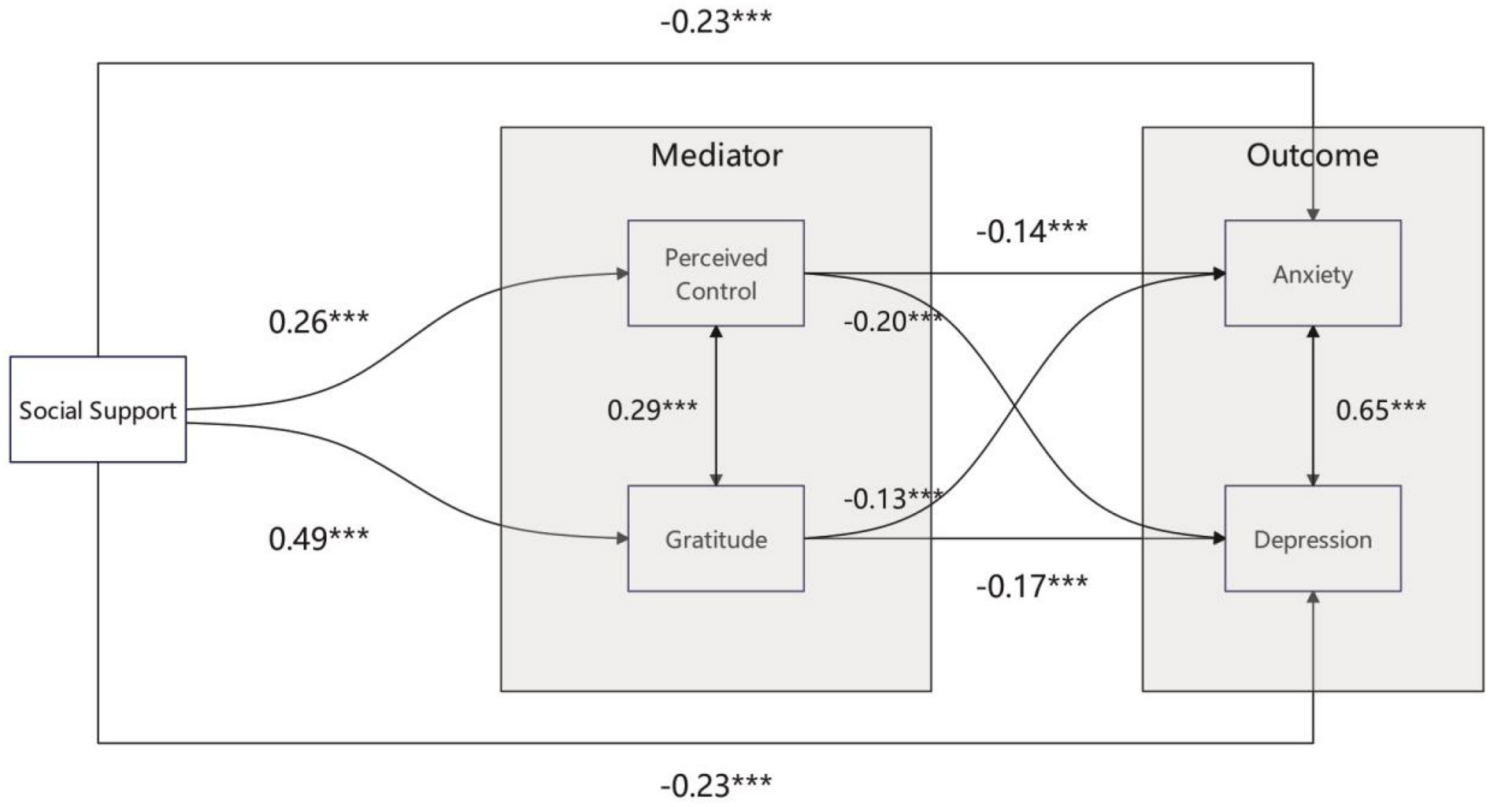
Structural equation model illustrating the direct and indirect pathways linking social support to depression and anxiety symptoms via perceived control and gratitude among Kenyan adolescents. Standardized path coefficients are shown. All paths are statistically significant at *p* < .001.

Structural equation modeling was used to test the hypothesized parallel mediation model, with social support as the predictor, perceived control and gratitude as mediators, and depression and anxiety symptoms as outcomes (Figure 1). All key parameter estimates are reported in Table 2.

**Table 2:** Comparison of unstandardized (B) and standardized (β) coefficients for indirect and total effects between base and control models.

Effect pathway	Base model				Control model (controlling for age and gender)			
	B	SE	95% CI	*β*	B	SE	95% CI	β
Indirect: Social Support → Perceived Control → Depression	−0.02***	0.003	[-0.07, −0.03]	-0.05***	-0.02***	0.003	[-0.02, −0.01]	−0.05***
Indirect: Social Support → Perceived Control → Anxiety	−0.01***	0.003	[−0.05, −0.02]	−0.04***	−0.01***	0.003	[−0.02, −0.01]	−0.03***
Indirect: Social Support → Gratitude → Depression	−0.03***	0.007	[−0.11, −0.06]	−0.08***	−0.03***	0.007	[−0.04, −0.01]	−0.08***
Indirect: Social Support → Gratitude → Anxiety	−0.02***	0.007	[−0.09, −0.04]	−0.06***	−0.02***	0.007	[−0.03, −0.01]	−0.07***
Total Effect: Social Support → Depression	−0.13***	0.011	[−0.15, −0.10]	−0.36***	−0.13***	0.011	[−0.15, −0.10]	−0.36***
Total Effect: Social Support → Anxiety	−0.11***	0.011	[−0.13, −0.09]	−0.33***	−0.11***	0.011	[−0.13, −0.09]	−0.33***

Note: *N* = 1,674. B, unstandardized coefficient; SE, standard error; 95% CI, 95% confidence interval for B; β, standardized coefficient. Control model includes age and gender as covariates. PCS, perceived control.

Confidence intervals are based on robust standard errors (MLR estimation). Significance levels are based on *p*-values associated with the z-statistic for each estimate.

*** *p* < .001.

Social support significantly predicted higher perceived control (*β* = 0.26, *p* < .001) and greater gratitude (*β* = 0.49, *p* < .001). In turn, both perceived control (*β* = −0.20 for depression, *β* = −0.14 for anxiety, both *p* < .001) and gratitude (*β* = −0.17 for depression, *β* = −0.13 for anxiety, both *p* < .001) were associated with lower levels of depression and anxiety symptoms. Importantly, social support also showed significant direct effects on both depression (*β* = −0.23, *p* < .001) and anxiety (*β* = −0.23, *p* < .001) even after accounting for the mediators.

Significant positive covariances were observed between perceived control and gratitude (*β* = 0.29, *p* < .001), and between depression and anxiety symptoms (*β* = 0.65, *p* < .001).

### Indirect and total effects

As shown in Table 2, all hypothesized indirect effects were statistically significant. Specifically, social support was associated with lower depression and anxiety symptoms via both perceived control (*β* = −0.05 and −0.04, respectively) and gratitude (*β* = −0.08 and −0.06, respectively), all *p* < .001. The total effects of social support on depression (*β* = −0.36, *p* < .001) and anxiety (*β* = −0.33, *p* < .001) were also significant, underscoring the robust protective role of social support on adolescent mental health.

These results provide empirical support for the mediating roles of both perceived control and gratitude in the relationship between social support and mental health outcomes. This suggests that interventions aimed at enhancing social support may be especially effective if they also foster greater perceived control and gratitude among adolescents.

### Sensitivity analyses with covariates

To examine the robustness of the findings, the mediation model was re-estimated controlling for age and gender. The pattern and magnitude of the main effects remained highly consistent (see Table 2). In these analyses, being male was associated with lower depression and anxiety symptoms, whereas older age was associated with higher depression and anxiety, which is consistent with established trends in adolescent mental health. Notably, the indirect and total effects of social support via perceived control and gratitude remained significant and nearly unchanged, demonstrating the stability of the mediation model across demographic groups.

## Discussion

This study investigated the complex interplay between social support, perceived control, gratitude, and mental health outcomes (depression and anxiety symptoms) among Kenyan adolescents. The central aim was to move beyond direct associations and explore an integrated model where the protective effects of social support are channeled through the parallel psychological resources of perceived control and gratitude. The findings largely affirmed this model, revealing that social support not only directly predicted lower internalizing symptoms but also operated indirectly by fostering both a sense of agency and an appreciative disposition in adolescents.

### An integrated model of psychosocial resilience in Kenyan adolescents

The current research offers a more holistic understanding by demonstrating that social support's benefits are not monolithic but are actualized through multiple, co-occurring psychological pathways. The significant mediational roles of both perceived control (*β* = −0.05 for depression, *β* = −0.04 for anxiety via this path) and gratitude (*β* = −0.08 for depression, *β* = −0.06 for anxiety via this path) underscore a synergistic process. Social support appears to simultaneously bolster adolescents’ cognitive belief in their capacity to influence outcomes (32) and cultivate their affective capacity to recognize and appreciate the good in their lives (33). This dual enhancement—of agency and appreciation—likely contributes to a more robust form of psychological resilience than if only one pathway were operative. For instance, an adolescent who feels supported may develop greater perceived control over academic challenges (34), while concurrently feeling more grateful for the educational opportunities and relational support they receive, both of which buffer against stress and promote well-being (35).

This integrated perspective extends the traditional stress-buffering hypothesis. While social support can directly mitigate the impact of stressors, our findings suggest it also proactively builds internal psychological capital. The positive association between social support and perceived control (*β* = 0.26) and an even stronger link with gratitude (*β* = 0.49) indicates that supportive environments are fertile ground for developing these key psychological assets. The significant covariance between perceived control and gratitude (*β* = 0.29) further suggests these resources are not independent but may be mutually reinforcing components of a positive psychological system nurtured by social support. An adolescent feeling a sense of control might be more open to recognizing and appreciating supportive actions, and conversely, a grateful disposition might enhance one's belief in the availability of resources and personal efficacy.

### Cultural nuances and the significance of the integrated model in Kenya

Examining this integrated model within the Kenyan context is particularly illuminating, as it moves beyond the typical focus on WEIRD societies (26) and offers a more globally representative understanding of adolescent mental health. The strength of the identified pathways is deeply resonant within the Kenyan socio-cultural landscape. For instance, the concept of social support aligns with the enduring ethos of “Harambee” (a Swahili term for “all pull together”), which continues to shape community-based social capital and collective action in modern Kenya (36). In such a collectivist context, where well-being is often perceived through a communal lens, social support is not merely a personal resource but a fundamental aspect of the social fabric, making its influence on mental health particularly potent (37). The exceptionally strong path from social support to gratitude (*β* = 0.49) may also be culturally inflected. Here, gratitude likely extends beyond a personal feeling to encompass a relational sense of reciprocity, a concept central to African worldviews like Ubuntu, which emphasizes interconnectedness and frames well-being as inherently relational. Similarly, the focus on academic perceived control is highly relevant in a context where education is widely viewed as the primary vehicle for upward social mobility, a belief that strongly shapes the aspirations and pressures faced by young people. This culturally-attuned model, which holds even after controlling for age and gender, addresses calls for more nuanced research on mental health correlates in Kenyan youth (38) and suggests that interventions will be most effective when they leverage these cognitive-agentic and affective-relational pathways in a culturally congruent manner.

The persistence of significant direct effects of social support on depression (*β* = −0.23) and anxiety (*β* = −0.23), even when accounting for perceived control and gratitude, is also an important aspect of this integrated understanding. It suggests that the benefits of social support extend beyond the cultivation of these specific psychological resources. Social support may also provide tangible aid, reduce direct exposure to certain adversities, or foster a general sense of belonging and security that has a direct soothing effect on distress, independent of an individual's level of perceived control or gratitude. This highlights that while perceived control and gratitude are crucial conduits, they form part of a broader supportive matrix.

### Practical implications

Our findings offer several actionable pathways for interventions aimed at promoting adolescent mental health in Kenya and similar contexts, suggesting a multi-level approach targeting schools, families, and policymakers. The integrated model indicates that strengthening social support is a critical first step, which in turn fosters the psychological resources of perceived control and gratitude. Within schools, this can be achieved by implementing structured peer mentoring programs and providing teachers with mental health literacy training, while families can contribute by fostering open, non-judgmental communication. Building on this foundation of support, both environments can actively cultivate perceived control and gratitude. For instance, educators can adopt a “growth mindset” approach and help students set incremental academic goals, while parents can involve adolescents in age-appropriate decision-making to enhance their sense of agency. Key policy actions should include integrating Social and Emotional Learning (SEL) into the national curriculum to systematically build these psychological skills, mandating and funding the presence of trained counselors in all secondary schools, and launching public health campaigns to destigmatize mental health and disseminate supportive parenting practices on a national scale.

### Limitations

This study has several limitations that should be noted. The primary limitation is the cross-sectional design, which precludes definitive causal inference (39); while our model is theoretically grounded, the relationships observed could be bidirectional. Furthermore, the sample was drawn from four secondary schools, which may limit the generalizability of our findings to the broader population of Kenyan adolescents (40), such as those not in school or in different regions. Third, our model included perceived control and gratitude, but other psychological factors like hope or optimism might also serve as important mediators. Future longitudinal research is essential to establish the temporal and causal nature of these pathways and to validate these findings in more diverse adolescent populations. In addition, while the sample was drawn from diverse school types (boys’ boarding, girls’ boarding, girls’ day, and mixed day) to enhance generalizability, we did not conduct a comparative analysis to test for model invariance across these groups due to the unequal distribution. It is possible that the strength of the pathways between social support, its mediators, and mental health outcomes differs by school environment (e.g., single-gender vs. mixed-gender). Future research could use multi-group structural equation modeling to explore these potentially important differences with appropriate data.

## Conclusion

This study offers novel insights and robust evidence regarding the mechanisms through which social support influences the mental health (specifically depression and anxiety symptoms) of Kenyan adolescents. The findings clearly demonstrate that social support not only directly alleviates adolescent depression and anxiety but, crucially, also indirectly promotes mental health by concurrently fostering two key psychological resources: perceived control and gratitude. These results carry significant theoretical and practical implications. Theoretically, this research validates and extends our understanding of the protective mechanisms of social support within a non-Western cultural context, highlighting the synergistic role of cognitive factors (perceived control) and positive affective traits (gratitude). From a practical standpoint, the findings suggest that interventions aimed at improving the mental health of Kenyan adolescents should not only focus on strengthening their social networks and available support but also actively design and integrate strategies to enhance their perceived control and cultivate gratitude.

## Data Availability

Publicly available datasets were analyzed in this study. This data can be found here: https://doi.org/10.1016/j.dib.2023.109082.
